# Computational screening of appealing perspectives of indium-based halide double perovskites In_2_AgSbX_6_ (X = Cl, Br, and I) for energy harvesting technologies

**DOI:** 10.1039/d5ra00242g

**Published:** 2025-04-09

**Authors:** Ahmad Ayyaz, M. Zaman, Hanof Dawas Alkhaldi, H. Irfan Ali, Imed Boukhris, S. Bouzgarrou, Murefah mana Al-Anazy, Q. Mahmood

**Affiliations:** a Centre for Advanced Studies in Physics, GC University Lahore 54000 Pakistan raiayyaz23@gmail.com; b Department of Physics, GC University Lahore 54000 Pakistan; c Department of Science and Technology, University College at Nairiyah, University of Hafr Al Batin (UHB) Nairiyah 31981 Saudi Arabia; d Department of Physics, Faculty of Science, King Khalid University P.O. Box 960 Abha Saudi Arabia; e Department of Physics, College of Science, Qassim University Buraidah 51452 Al-Qassim Saudi Arabia; f Laboratoire de Microélectronique et Instrumentation (UR03/13-04), Faculté des Sciences de Monastir Avenue de l'Environnement 5000 Monastir Tunisia; g Department of Chemistry, College of Sciences, Princess Nourah bint Abdulrahman University (PNU) P.O. Box 84428 Riyadh 11671 Saudi Arabia; h Department of Physics, College of Science, Imam Abdulrahman Bin Faisal University P.O. Box 1982 Dammam 31441 Saudi Arabia; i Basic and Applied Scientific Research Center, Imam Abdulrahman Bin Faisal University P.O. Box 1982 Dammam 31441 Saudi Arabia qmmustafa@iau.edu.sa

## Abstract

Halide double perovskites have attracted considerable attention for their potential use in solar cells and thermoelectric devices, as they are ecologically benign and possess band gap tunability. Herein, the stability, optoelectronic, and thermal transport characteristics of In_2_AgSbX_6_ (X = Cl, Br, and I) were examined using density functional theory (DFT). *Ab initio* molecular dynamics (AIMD) analysis was conducted, which verified the dynamic stability of In_2_AgSbX_6_ up to 700 K. The estimated elastic parameters further confirmed their mechanical stability. Through mechanical analysis, the asymmetric characteristics of In_2_AgSbX_6_ were revealed. The above-mentioned materials were ductile, validating their utilization in flexible or foldable technologies. Analyses of the electrical properties of In_2_AgSbCl_6_, In_2_AgSbBr_6_, and In_2_AgSbI_6_ showed indirect band gaps (*E*_g_) of 1.95 eV, 1.35 eV, and 0.78 eV, respectively. These electronic *E*_g_ values were ideal for solar cell applications. The lower effective masses and binding energies of excitons of In_2_AgSbCl_6_, In_2_AgSbBr_6_, and In_2_AgSbI_6_ than those of the perspective solar cell candidates CsPbI_3_ and Cs_2_AgBiBr_6_ provided evidence for their effectiveness as absorber layer materials. The optical analysis of the dielectric constant, absorption, reflection, and loss demonstrated higher absorption, lower reflection, and minimal energy loss within the visible and ultraviolet spectra. The thermal transport features were analyzed for various temperatures up to 600 K and chemical potentials. In_2_AgSbCl_6_, In_2_AgSbBr_6_, and In_2_AgSbI_6_ demonstrated p-type nature, higher Seebeck coefficient, and *ZT* values of 0.75, 0.77, and 0.76, respectively. Thus, In_2_AgSbCl_6_, In_2_AgSbBr_6_, and In_2_AgSbI_6_ possessed feasible characteristics for applications in solar cells and thermal energy transformation, demonstrating that they can be utilized in future energy harvesting technologies.

## Abstract 2

The indirect band gaps (*E*_g_) of 1.95 eV, 1.35 eV, and 0.78 eV and primary absorption *α*(*ω*) peaks in the visible region suggest that the In_2_AgSbX_6_ (X = Cl, Br, and I) is suitable for solar cells.

## Graphical abstract

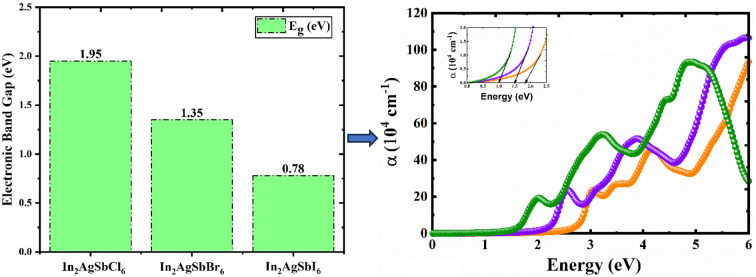

## 1. Introduction

Energy is an essential aspect for the sustenance of contemporary civilization, and it facilitates the ongoing socioeconomic and commercial improvement and evolution of future generations.^1^ The rapid exhaustion of fossil resources and escalating energy needs threaten the occurrence of a global energy calamity. Considering this possible energy shortage, scientists are compelled to identify cost-effective and environmentally sustainable renewable energy sources. Photovoltaics is a promising method for addressing future energy requirements.^2,3^ In recent times, perovskite solar cells (PSCs) containing a lead halide in an organic/inorganic mixture have garnered significant interest and attained a power conversion efficiency (PCE) of over 26.1%.^4^ Nevertheless, the inadequate chemical stability of these halides and the poisonous nature of lead are hindering the technological advancement of these photovoltaic cells. Inorganic-only PSCs exhibit excellent stability at elevated temperatures and moist conditions, effectively capturing light throughout the visible range of the spectrum.

Currently, emphasis is placed on compounds with formulations of A_2_M^+^M^3+^X_6_, which have garnered significant interest owing to their tremendous stability and superior solar energy generation capacity.^5,6^ The PCE of double perovskite (DP) solar cells has achieved an incredible theoretical value of approximately 30% in the recent past,^7^ suggesting that they are viable alternatives to silicon cells, which are currently used in commercial applications.^8^ Moreover, halide replacement in DPs has led to substantial progress in materials discovery, and accordingly, DPs are recognized as a compelling technology for the future development of renewable energy technologies.^9^ A wide variety of experiments and computational studies have been conducted to elucidate the physical and inherent defect characteristics of DP derivatives. Zhang *et al.* studied hydrogenated Cs_2_AgBiBr_6_, revealing a band gap (*E*_g_) reduction from 2.18 eV to 1.64 eV, high carrier mobility, and 6.37% ECE.^10^ Moreover, Cs_2_SnI_6_, which exhibits remarkable performance and an appropriate bandgap of 1.6 eV, is an extremely promising light-harvesting material. The power conversion efficiency of Cs_2_SnI_6_ was 8%.^11^ García-Espejo *et al.* synthesized Cs_2_AgSbBr_6,_ which showed greater stability and an *E*_g_ of 1.93 eV.^12^ Bhorde *et al.* investigated Rb_2_AgBiI_6_ and revealed its semiconductor nature with a 1.98 eV band gap.^13^ These limited studies on halide DPs have motivated computational scientists to predict DPs and suggest materials with exceptional characteristics to experimentalists.

Therefore, density functional theory (DFT) is a key computational tool for predicting the crystal structure and physical properties of semiconductor materials.^14^ Alotaibi *et al.* estimated the energy harvesting potential of Cs_2_AgBi(Cl/Br/I)_6_.^15^ Hnuna *et al.* examined the band gap and optical performance of DPs Rb_2_AgIn(Cl/Br/I)_6_, which showed visible absorption and p-type semiconductors.^16^ In addition, Cs_2_NaInBr_6_ and Cs_2_NaInI_6_ exhibit superior stability and are suitable for wasted thermal energy.^17^ Rb_2_InSb(F/Cl/Br/I)_6_ has been used to elucidate direct *E*_g_ and has shown exceptional optical attributes for Br and I-based compounds.^18^ Moreover, several other combinations based on DFT explorations^19–22^ require further validation by experimental researchers. In addition, recent studies on indium-based DP combinations have shown their significance and effectiveness.^23,24^ Recently, new single perovskites InGeF_3_, InGeCl_3_, and InGeI_3_ are efficient for photovoltaics and thermal energy conversion applications.^25^ Consequently, this study motivated us to predict the photovoltaic and thermoelectric potentials of innovative indium-based DP combinations.

The present investigation focused on the performance analysis of DP In_2_AgSbX_6_ (X = Cl, Br, and I) combinations based on optical and thermoelectric analysis. The stability under cubic arrangement and under mechanical conditions was also investigated, which is crucial for various technological applications. To the best of our knowledge, there has been no written research on the studied In_2_AgSbX_6_ (X = Cl, Br, and I) DP combinations in literature. This report presents a comprehensive DFT-based examination of In_2_AgSbCl_6_, In_2_AgSbBr_6_, and In_2_AgSbI_6,_ accompanied by a full explanation of the relevant DP compounds, highlighting the significance of the analyzed DP combinations for future applications in photovoltaics and various other power generation technologies.

## 2. Computational methodology

This work employed DFT by deploying the WIEN2k code^26^ to assess the optical and thermoelectric analysis of In_2_AgSbCl_6_, In_2_AgSbBr_6_, and In_2_AgSbI_6_. This code employs the full potential linearized augmented plane wave (FP-LAPW) method.^27^ The optimization procedure was conducted for the simulated cubic frameworks to obtain the optimal lattice constants (*a*_0_) and structural parameters. Following the verification of the structural characteristics, self-consistent-field (SCF) computations were conducted to determine the electronic characteristics and associated aspects. The Generalized Gradient Approximation (GGA) established by Perdew, Burke, and Ernzerhof (PBE)^28^ was employed to address compositional considerations. To rectify the recognized band gap underestimation, the Tran–Blaha–modified Becke–Johnson technique (TB-mBJ) was employed.^29^ The maximum angular momentum value, *l*_max_, is established at 10 and *G*_max_ was fixed at 14. The muffin tin radii (*R*_MT_) values (in atomic units) for the specified components are as follows: 2.1 for In, 2.0 for Ag, 2.0 for Sb, and 2.0 for Cl or Br. The *R*_MT_ × *K*_max_ = 8 was selected, accompanied by a binding energy value of −6 Ry, where *R*_MT_ is the muffin-tin radius of the smallest constituent of the examined DP compounds. The optical and thermoelectric aspects were determined using TB-mBJ with a dense k-mesh of 10 × 10 × 10. Additionally, some thermoelectric (TE) characteristics have been calculated using the BoltzTraP code, which uses semiclassical Boltzmann theory to understand TE aspects and the relationship with temperature and chemical potential of the TE properties. The constant relaxation time approximation (CRTA) was employed to compute the TE parameters, with constant relaxation time (*τ*) set at 10^−14^ s.

## 3. Results and discussion

### 3.1. Structural properties and stability

**Fig. 1:**
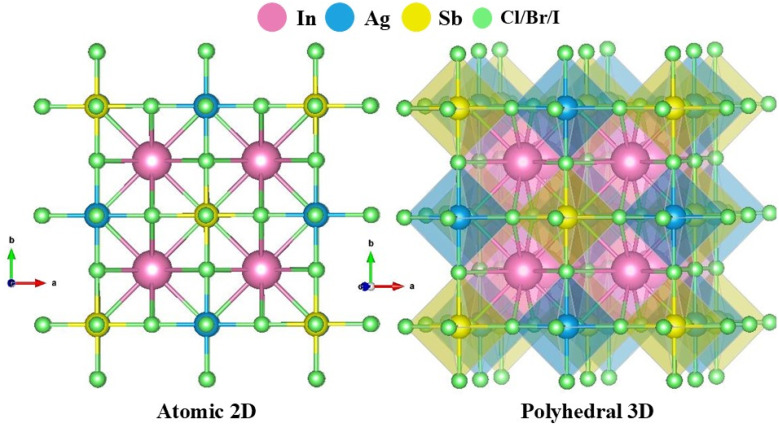
Atomic visualization of the cubic structure for DP compounds of In_2_AgSbX_6_ in 2D and 3D polyhedral view.

**Fig. 2:**
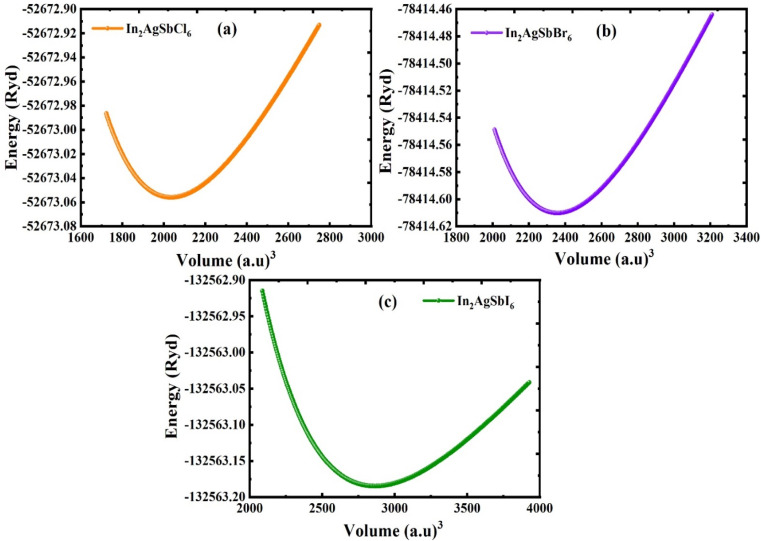
Varying energy *versus* volume curve obtained by optimizing the structures of (a) In_2_AgSbCl_6,_ (b) In_2_AgSbCl_6,_ and (c) In_2_AgSbI_6_.

The structural characteristics were analyzed to determine the specifics of the arrangement and to evaluate the operational variations in the features of the examined substances. The compounds In_2_AgSbX_6_ (X = Cl, Br, and I) possess an A_2_M^+^M^3+^X_6_ crystalline structure and are classified under the space group *Fm*3̄*m*, with a space group number of 225. The structural configuration of the In_2_AgSbX_6_ perovskite with the cubical framework is illustrated in Fig. 1. The total energy of In_2_AgSbX_6_ was reduced by optimizing the changes in the unit cell volume, as shown in Fig. 2. This facilitates the identification of ground state characteristics. In proximity to the volume at the ground state (*V*_0_), the framework exhibits stability in the equilibrium or ground state. Significantly expanding the volume beyond *V*_0_ may decrease energy as the structure moves toward greater stability. As the substance approaches equilibrium, the potential energy (PE) decreases, and the volume slightly increases. At equilibrium volume, the energy reaches its minimum value (*E*_0_), which reflects the most stable configuration of the DP compound under scrutiny. When the volume exceeds *V*_0_, the energy starts to increase once more because the substance has been elongated beyond its stable configuration, resulting in a heightened PE due to increased atomic separation. The obtained lattice constant (*a*_0_) and equilibrium parameters (*V*_0_ and *E*_0_) are presented in Table 1. A notable difference in *a*_0_, *V*_0_, and *E*_0_ is observed when In_2_AgSbCl_6_ is interchanged with In_2_AgSbBr_6_ and In_2_AgSbI_6_, which are replaced in the compounds. The variation in the dimensions of the halides results in a regular shift in the lattice parameters (*a*_0_), exemplifying the “octahedral impact,” which dramatically affects the volume of the unit cell.^30^ The *a*_0_ values for In_2_AgSbX_6_ (X = Cl, Br, and I) are consistent with those of similar materials such as Rb_2_AgSbX_6_ (X = Cl, Br) and Cs_2_AgSbX_6_ (X = Cl, Br, and I), as displayed in Table 1. The ionic radius of In is 0.8 Å, which is less than that of Rb (1.52 Å) and Cs (1.67 Å). Therefore, In_2_AgSbX_6_ had relatively lower *a*_0_ values than Rb_2_AgSbX_6_ and Cs_2_AgSbX_6_. This examination emphasizes the correlation between halide substitution and structural alterations, illustrating the significant implications of Cl/Br/I size on the entire crystal framework and the long-term stability of these substances.

**Table 1:** Depicted structural characteristics and stability governing parameters of In_2_AgSbX_6_

Compounds	*a*_0_ (Å) this work	*a*_0_ (Å) other works	*V*_0_, (a.u.)^3^	*E*_0_ (Ry)	*B* (GPa)	*τ*	*E*_f_ (eV per atom)
In_2_AgSbCl_6_	10.64	10.71 (ref. 33), 10.77 (ref. 34)	2036.9395	−52673.0561	29.73	0.94	−1.97
In_2_AgSbBr_6_	11.18	11.26 (ref. 33), 11.12 (ref. 34)	2359.4342	−78414.6102	25.13	0.94	−1.55
In_2_AgSbI_6_	11.93	11.99 (ref. 34)	2865.6652	−132563.1825	19.02	0.95	−1.32

The calculation of the tolerance factor confirms the integrity or stability of cubic DP compounds, which can be calculated from the ionic radii as follows:1
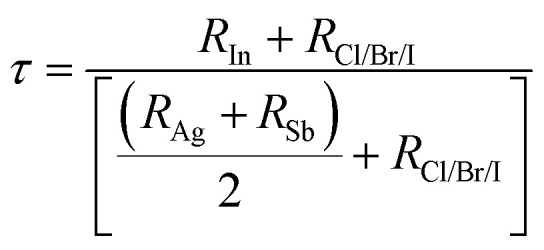


The resulting values (Table 1) vary from 0.81 to 1.11 for the designated materials, confirming that they remain stable in the cubic framework.^31^ Furthermore, to computationally assess the feasibility of synthesis and evaluate the resilience of these DP substances against spontaneous disintegration into alternative binary or elemental segments, the formation energy (*E*_f_) is determined from the enthalpy, as described in ref. 32:

2 *E*_f_ = *E*_In_2_AgSbX_6__ − (2*E*_In_ + *E*_Ag_ + *E*_Sb_ + 6*E*_X_)

The ascertained *E*_f_ values are shown in Table 1. The negative values signify that In_2_AgSbX_6_ (X = Cl, Br, and I) is chemically stable, indicating that these compounds can be synthesized practically under atmospheric circumstances.

**Fig. 3:**
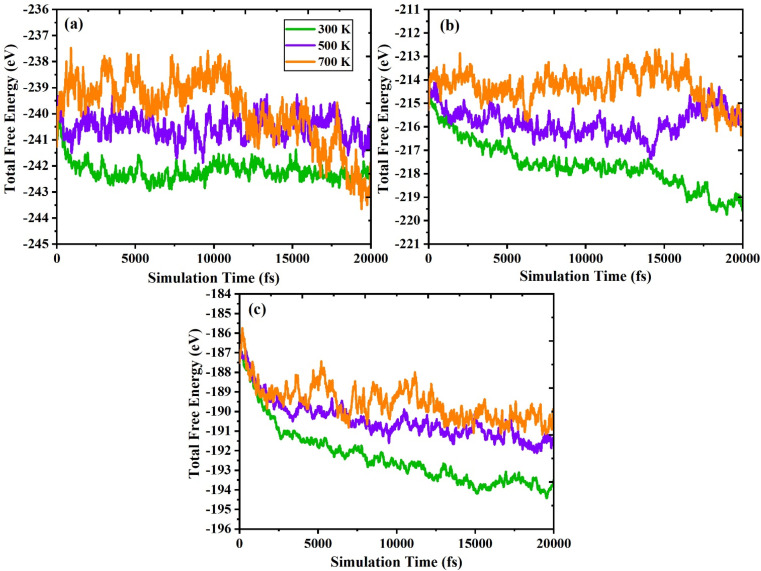
AIMD plots showing total free energy (eV) *versus* simulation time (fs) for (a) In_2_AgSbCl_6,_ (b) In_2_AgSbBr_6,_ and (c) In_2_AgSbI_6_.

In addition, we executed *ab initio* molecular dynamics (AIMD) calculations at 300 K, 500 K, and 700 K to confirm the stability of In_2_AgSbCl_6_, In_2_AgSbBr_6_, and In_2_AgSbI_6_. The canonical ensemble (*NVT*) with an interval period of 1.0 femtosecond (fs) and the Nosé thermostat were employed to perform the computations. The results are shown in Fig. 3(a–c)3, and the computation time was up to 20 000 picoseconds. The material's stability is demonstrated by the fact that, while considering both the total energy and the simulation duration, the AIMD research revealed energy variations around an equilibrium average value. However, suppose the total energy levels are consistently rising or falling. In this case, the results might indicate that the material is unstable because of an abrupt shift in phase or a change in its chemical structure. In addition, due to thermal vibrations within the structure, a small loss of energy occurs due to temperature variations between 300 K and 700 K. Regardless, In_2_AgSbCl_6_, In_2_AgSbBr_6_, and In_2_AgSbI_6_ show a stable mean total energy value for temperature fluctuations. The fact that these halides remain stable indicates that neither phase nor structural breakdown has evolved significantly. Thus, the double perovskites In_2_AgSbCl_6_, In_2_AgSbBr_6_, and In_2_AgSbI_6_ exhibit dynamic stability and may be used for future technological applications.

### 3.2. Elastic properties

**Fig. 4:**
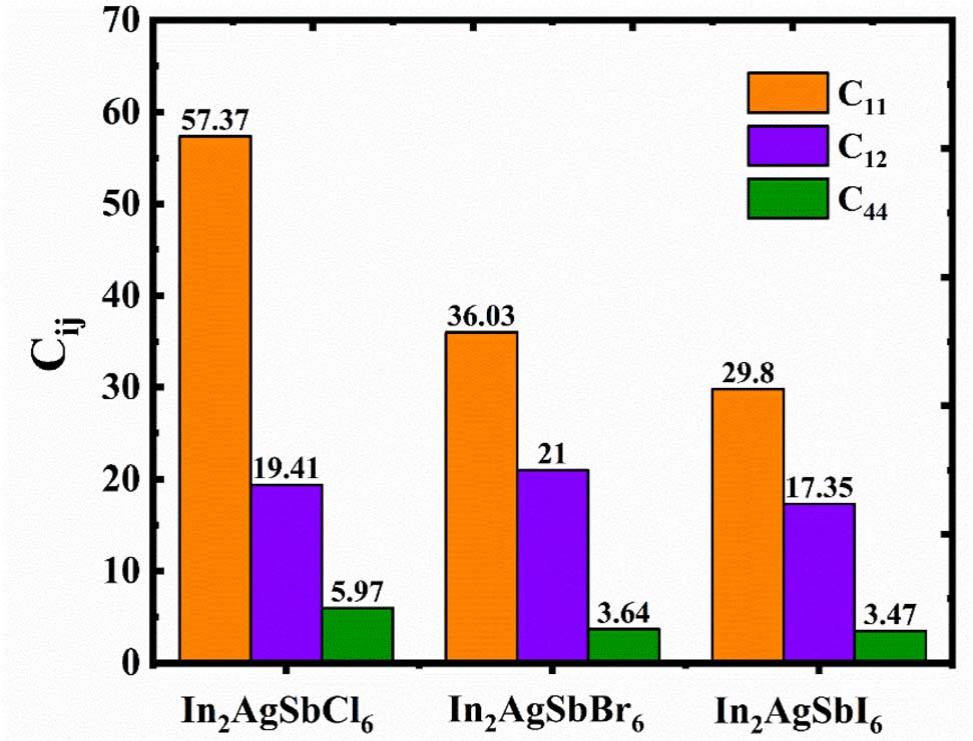
Calculated elastic constants (*C*_*ij*_) obtained for In_2_AgSbX_6_.

**Fig. 5:**
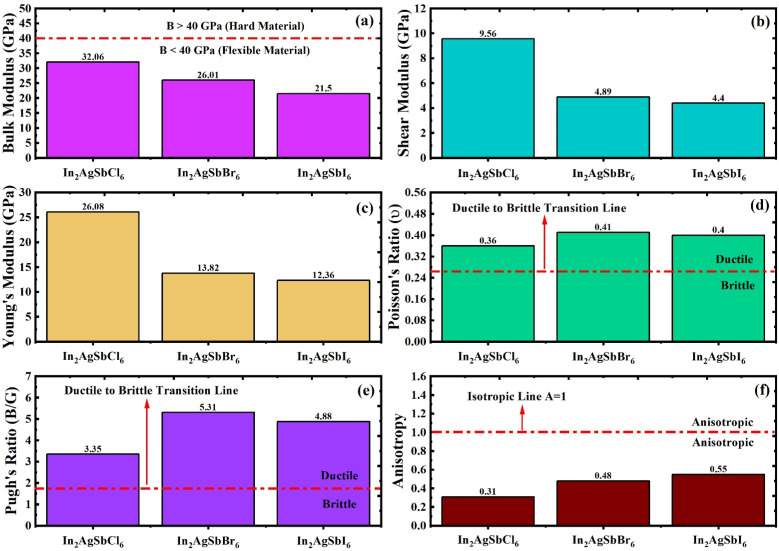
Elastic parameters obtained from *C*_*ij*_ values (a) *B* (b) *G* (c) *Y* (d) *ν* (e) B/G (f) *A*.

A thorough analysis of the structure-related characteristics yields an understanding of the mechanical behavior of substances.^35^ The atomic configuration inside the framework is crucial for estimating the framework's susceptibility to external pressures and thus determines its mechanical stability. The evaluation of this characteristic is essential for device manufacturing and engineering applications. We determined the elastic constants (*C*_*ij*_) of the specified DP materials using the volume and energy method while preserving deformation tetrahedrally and rhombohedrally inside the cubic flexible framework.^36,37^ The estimated *C*_*ij*_ (*C*_11_, *C*_12_, and *C*_44_) for In_2_AgSbCl_6_, In_2_AgSbBr_6_, and In_2_AgSbI_6_ halide DP materials (Fig. 4) were positive and uphold the Born stability criteria.^38^ The computed *C*_*ij*_ offers perspectives on the stability under mechanical forces and enables the examination of diverse elastic properties. Several other factors, including bulk (*B*), shear (*G*), and Young's (*Y*) elastic moduli, are obtained from *C*_*ij*_ using mathematical relationships documented in previous studies,^39^ with the results presented in Fig. 5(a–c).

Young's modulus indicates the degree of rigidity of DP compounds, indicating their capacity to hold up to distortion during horizontal stretching or compressive stresses. In contrast to Young's modulus, which pertains to rigidity or hardness, the bulk modulus emphasizes the ability to compress a material, with elevated values signifying increased resistance to volumetric changes. The shear modulus (*G*), which denotes the ability to endure plastic deformation, suggests that lower *G*-values enable less endurance against plastic deformation in DP compounds.^40^ According to the data presented in Fig. 5(a), In_2_AgSbCl_6_ exhibited a higher *B* value of 32.06, which is considerably greater than the *B* values of In_2_AgSbBr_6_ (26.01) and In_2_AgSbI_6_ (21.5). Although In_2_AgSbCl_6_, In_2_AgSbBr_6_, and In_2_AgSbI_6_ reveal fewer values than 40 GPa (for hard materials)^41^ and show a flexible nature, In_2_AgSbCl_6_ is superior in withstanding volumetric changes than In_2_AgSbBr_6_ and In_2_AgSbI_6_. Because of these characteristics, In_2_AgSbCl_6_ is a promising option for use in thin-film solar cell manufacturing, where flexibility and stress resilience are crucial.^42^ Moreover, In_2_AgSbCl_6_ shows higher values of *G* (9.56) and *Y* (26.08) than In_2_AgSbBr_6_ and In_2_AgSbI_6_, as demonstrated in Fig. 5(b) and (c), respectively, indicating higher endurance against plastic deformation and hardness. This higher plasticity indicates that In_2_AgSbCl_6_ is comparatively more resistant to plastic deformation than In_2_AgSbBr_6_ and In_2_AgSbI_6_, making it suitable for foldable devices. Additionally, the higher rigidity of In_2_AgSbCl_6_ is suitable for systems requiring higher structural stability, such as resistance to fracture under applied pressure.

Furthermore, the analysis of the ductile nature of materials further confirms their suitability for use in flexible optoelectronics and foldable thin films for solar cell applications, which can be determined by elastic features.^43^ Poisson's ratio (*ν*) showcases insights into a material's ductility and flexibility, which is elucidated by values ranging from 0.26 to 0.42, below which materials are referred to as brittle. The values of *ν* for In_2_AgSbCl_6_, In_2_AgSbBr_6_, and In_2_AgSbI_6_ were 0.36, 0.41, and 0.40, respectively, as shown in Fig. 5(d). Moreover, the ductile or brittle features of In_2_AgSbCl_6_, In_2_AgSbBr_6_, and In_2_AgSbI_6_ may be evaluated more thoroughly using Pugh's ratio, which is represented as B/G. Fig. 5(e) indicates that the Pugh ratio for In_2_AgSbCl_6_, In_2_AgSbBr_6_, and In_2_AgSbI_6_ exceeds the crucial value of 1.75, thereby affirming the ductile characteristics of these perovskites. This ductility is essential for systems that require materials capable of enduring mechanical stresses without breaking, such as flexible technologies like solar cells.

### 3.3. Mechanical anisotropy

Mechanical anisotropy is a crucial characteristic that significantly affects several physical characteristics. It significantly influences phenomena such as permanent deformation and fracture formation in crystals, as well as the mechanical characteristics of materials, especially their texture. The relevance of these consequences is significant in the field of engineering because comprehending the influence of mechanical anisotropy on the functionality of a substance is crucial for the creation of efficient and lasting structures. Diverse anisotropy indexes are employed to determine the orientation dependency of a material's characteristics. A commonly used metric for purposes such as the anisotropic parameter (*A*) quantifies the disparity in binding strength among atoms situated in different crystalline axes. This allows scientists and technologists to modify the layout and functionality to meet particular requirements. A comprehensive understanding of these anisotropic features can enhance material effectiveness, improve structural durability, and ensure the longevity and safety of materials. Due to the symmetry of In_2_AgSbCl_6_, In_2_AgSbBr_6_, and In_2_AgSbI_6_ in cubical configuration, *A* was determined using the following relation:3
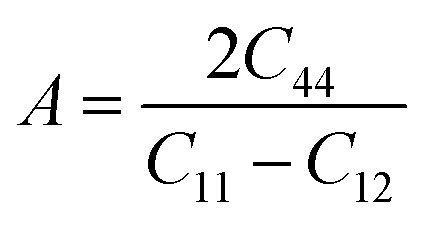


Fig. 5(f) presents the A values, which are below the isotropic line (*A* = 1), indicating that In_2_AgSbCl_6_, In_2_AgSbBr_6_, and In_2_AgSbI_6_ are anisotropic DP compounds.^44^ The *A* values indicate that In_2_AgSbCl_6_ is highly anisotropic compared to In_2_AgSbBr_6_ and In_2_AgSbI_6_, affirming that In_2_AgSbCl_6_ is more durable and long-lasting.

**Fig. 6:**
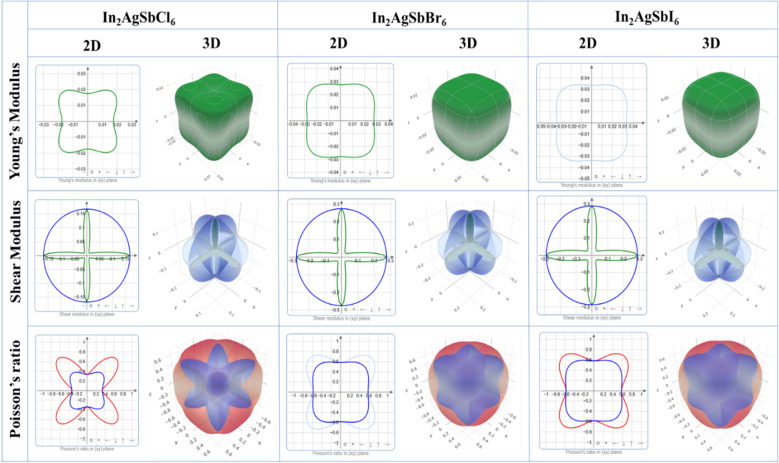
Graphical assessment of anisotropy showing 2D and 3D figures.

To illustrate the degree of anisotropy of elastic moduli in In_2_AgSbCl_6_, In_2_AgSbBr_6_, and In_2_AgSbI_6_ DP compounds, three-dimensional surface designs (3D contours) and their two-dimensional figures (2D contours) were generated. The graphic representations were generated using the ELATE tool,^45^ which uses the estimated *C*_*ij*_ values. In an isotropic crystallized material, such representations are often shown to be exact spheres in three-dimensional space. Nonetheless, for anisotropic substances like In_2_AgSbCl_6_, In_2_AgSbBr_6_, and In_2_AgSbI_6_, the three-dimensional surfaces diverge from a sphere-like configuration, signifying varying mechanical qualities along distinct crystalline orientations.^46^Fig. 6 shows the 2D and 3D anisotropic visualizations for Y and G (in GPa) and *ν* of the In_2_AgSbCl_6_, In_2_AgSbBr_6_, and In_2_AgSbI_6_ compositions, illustrating the degree of mechanical anisotropy. Anisotropy is evident in the irregular shape of the 3D surfaces, demonstrating the directional sensitivity of the elastic characteristics inside the crystalline structure.

This investigation entails the computation and graphical representation of the lowest and highest estimated values of *Y* and *G* (in GPa) and *ν* of the In_2_AgSbCl_6_, In_2_AgSbBr_6_, and In_2_AgSbI_6_ in various orientations, as shown in Table 2. The descending order of anisotropy for these parameters is In_2_AgSbCl_6_ > In_2_AgSbBr_6_ > In_2_AgSbI_6_. Hence, the hierarchy of the values indicates that In_2_AgSbCl_6_ has the greatest degree of anisotropy, followed by In_2_AgSbBr_6_, and finally In_2_AgSbI_6_. Graphical and numerical assessments are essential for understanding the elastic characteristics of In_2_AgSbCl_6_, In_2_AgSbBr_6_, and In_2_AgSbI_6_ with different crystal lattice orientations. These findings are crucial in technology and development for the customization and enhancement of materials' mechanical characteristics in particular applications, thereby assuring their dependability and reliability under diverse stress circumstances.

**Table 2:** Numerical assessment of anisotropy showing lowest and highest values for In_2_AgSbCl_6_, In_2_AgSbBr_6_, and In_2_AgSbI_6_

Parameters		In_2_AgSbCl_6_	In_2_AgSbBr_6_	In_2_AgSbI_6_
*Y* (Gpa)	*Y*_min_	0.017431	0.027755	0.033557
	*Y*_max_	0.029366	0.036733	0.044137
	*A*	1.685	1.323	1.315
G (Gpa)	*G*_min_	0.013172	0.033267	0.040161
	*G*_max_	0.1675	0.27473	0.28818
	A	12.72	8.258	7.176
*ν*	*ν*_min_	−0.95706	−0.95433	−0.94429
	*ν*_max_	−0.33833	−0.58285	−0.58221
	*A*	0.3535	0.6107	0.6166

### 3.4. Electronic properties

**Fig. 7:**
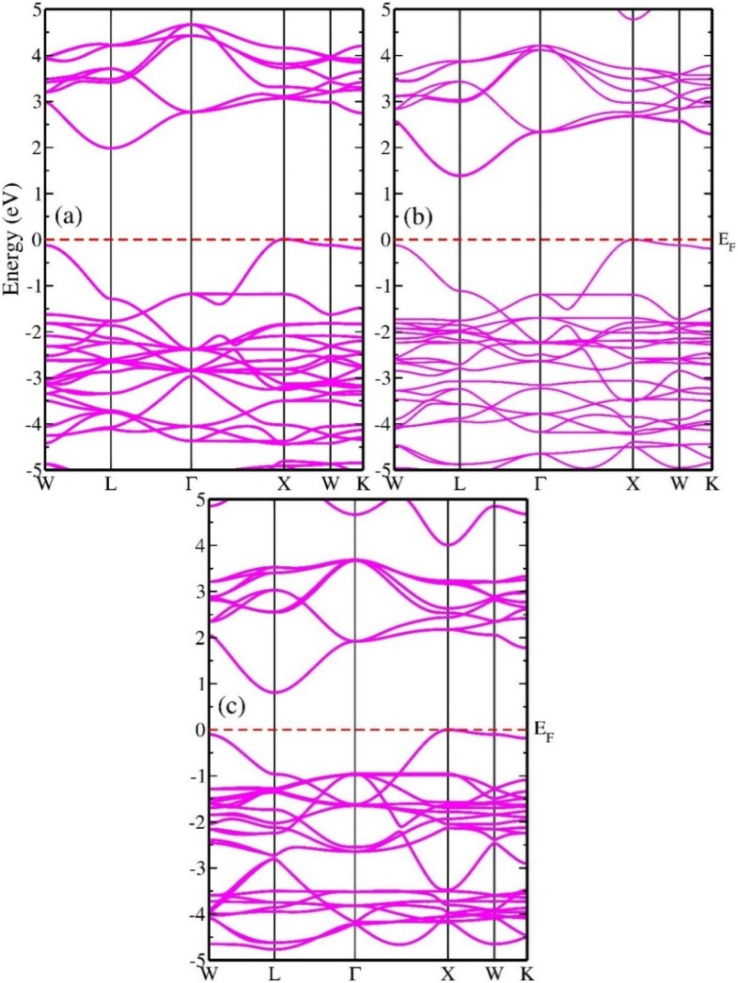
Electronic BS showing indirect *E*_g_ of (a) In_2_AgSbCl_6_, (b) In_2_AgSbBr_6_, and (c) In_2_AgSbI_6_.

In the analysis of electronic properties, estimation of the band structure (BS) is essential because it provides information on the distribution of the energy concentrations of electrons inside a substance. Before substances can be used in different technologies, their energy BS must be assessed to obtain a more comprehensive understanding of the materials. Thus, insulating substances, semiconductor components, and metallic compounds can be categorized according to their energy BS.^47^Fig. 7(a–c) show the band profiles of In_2_AgSbCl_6_, In_2_AgSbBr_6_, and In_2_AgSbI_6_, respectively, obtained using the mBJ approximation. There is a separation between the conduction and valence bands (CB and VB) because their energies are shifted from the Fermi level (*E*_F_), as visualized from the BS shown in Fig. 7. Additionally, Fig. 7 indicates that In_2_AgSbCl_6_, In_2_AgSbBr_6_, and In_2_AgSbI_6_ are semiconductors because their energy band gaps (*E*_g_) range from 0.7 to 2.0 eV. In_2_AgSbCl_6_ exhibits an indirect (*L*–*X*) *E*_g_ of 1.95 eV, and In_2_AgSbBr_6_ exhibits an indirect (*L*–*X*) *E*_g_ of 1.35 eV. For In_2_AgSbI_6_, the value of *E*_g_ was further reduced to 0.78 eV (*L*–*X*). The bandgap decreased when the halogen atom size increased from In_2_AgSbCl_6_ to In_2_AgSbI_6_, as shown in Fig. 7. As the lattice constant rises, the spacing between atoms will broaden. It follows that the coulombic interaction between the electrons in VB and the nucleus would become weaker, resulting in adjustments in the electronic states in CB closer to *E*_F_. Therefore, the minimal amount of energy needed to transform the constrained electrons in VB into electrons in CB allows them to move unhindered inside the material. The energy required to excite valence electrons from the VB to CB decreases as the separation between atoms increases, which is the primary requirement for efficient electronic transitions.^48^

Furthermore, the extracted BS and tunability of *E*_g_ indicate that In_2_AgSbCl_6_, In_2_AgSbBr_6_, and In_2_AgSbI_6_ can be used in innovative optical technologies such as photovoltaic and thermal energy conversion systems. The band structure configurations of materials with respect to the Fermi level may establish their characteristics exactly based on semiconducting theory. The chemicals included in this investigation have electrical band gap values similar to those of other perovskite-based substances that have been demonstrated in the literature; these values are shown in Table 3.

**Table 3:** Predicted effective mass of electrons and holes and exciton binding energy

Compounds	*E*_g_ (eV) this work	*E*_g_ (eV) other works	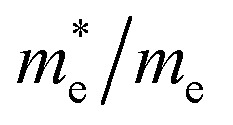	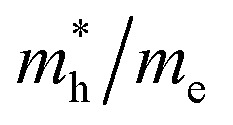	*ε*_1_(0)	*E* ^ex^_b_ (eV)
In_2_AgSbCl_6_	1.95	2.19 (ref. 33), 1.58 (ref. 34)	0.1401	0.1469	4.08	0.0584
In_2_AgSbBr_6_	1.35	1.56 (ref. 33), 1.16 (ref. 34)	0.1068	0.1034	4.67	0.0326
In_2_AgSbI_6_	0.78	0.53 (ref. 34)	0.0753	0.1153	6.88	0.0130

**Fig. 8:**
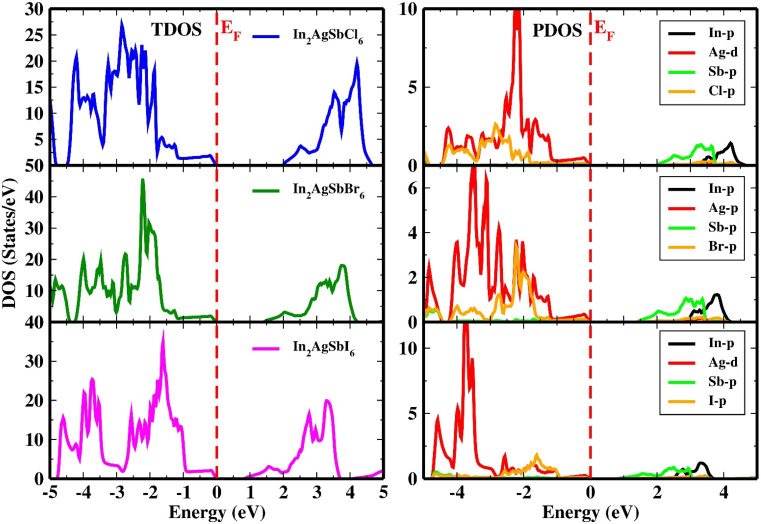
Orbital projected and total density of states for In_2_AgSbCl_6_, In_2_AgSbBr_6_, and In_2_AgSbI_6_.

The predicted densities of states of the atoms are shown in Fig. 8 to evaluate the contribution of atoms to the formation of VB and CB. Fig. 8 shows the orbital projected density of states (PDOS) and total density of states (TDOS) of In_2_AgSbCl_6_, In_2_AgSbBr_6_, and In_2_AgSbI_6_. From this analysis, it can be seen that the transition metal Ag's d-orbital occupies the states in the VB, whereas the p-states of Sb atoms characterize the CB. The *E*_F_ was located close to the VB, the highest point indicating the p-type nature of the materials. PDOS discovered that at *E*_F_, the d-orbital of the Ag atom at the maximum VB states and the p-states of Sb atoms at the CB minimum contributed the most. Due to the modification of In_2_AgSbCl_6_ with In_2_AgSbBr_6_ and In_2_AgSbI_6_, there is a shift in the electronic states in the CB above *E*_F_. Therefore, the substitution of halogen atoms causes a shift in the electronic states, thereby facilitating electronic transitions. The p-orbitals of the In also contribute to the generation of the CB in the compounds under study, with the contribution being slightly distant from *E*_F_ compared to the Sb-p. Moreover, the VB is also contributed by the p-orbital of Cl/Br/I, which is less than the Ag-d states. For all configurations, no hybridization was observed in the creation of energy levels at the *E*_F_ due to the energy gap. Therefore, the contributing atomic states in the VB and CB will have greater significance in assessing the electronic transitions for technological applications.

In addition, the effective mass is a crucial measure for assessing the optical and electrical features of substances, such as conductance and mobility. The movement of carriers is inversely proportional to the effective mass values of holes and electrons, which decrease with increasing effective mass values.^49^Eqn (4) facilitates the calculation of the effective masses by non-linear fitting of the BS curve.4
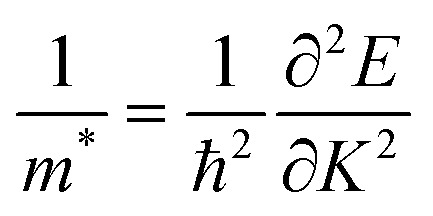


Additionally, the exciton binding energy (*E*^ex^_b_) was evaluated by utilizing the effective masses and dielectric constant at zero frequency *ε*_1_(0) by the given eqn (5). Additionally, the exciton binding energy is the amount of energy required to separate an exciton, which consists of two electrons and a hole, into free carriers of energy. In photovoltaic technologies, effective absorption and carrier collection are made possible by materials with low binding energies.5
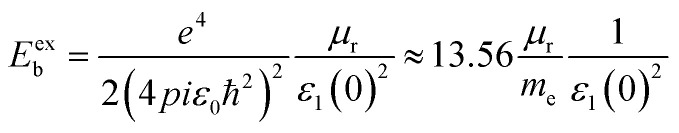


The effective masses and binding energies are listed in Table 3. The electrons and holes in In_2_AgSbCl_6_ have a higher effective mass than the electrons in In_2_AgSbBr_6_ and In_2_AgSbI_6_, suggesting that In_2_AgSbCl_6_ electrons are more inert or face greater movement resistance.^49^ Additionally, the values of *E*^ex^_b_ have shown that In_2_AgSbCl_6_, In_2_AgSbBr_6_, and In_2_AgSbI_6_ have lower exciton binding energies than CsPbI_3_ (0.068 eV),^50^ Cs_2_AgBiBr_6_ (0.268 eV),^51^ and Cs_2_AgBiBr_6_ (0.227 eV).^52^ This indicates that In_2_AgSbCl_6_, In_2_AgSbBr_6_, and In_2_AgSbI_6_ can serve as absorber layer materials in solar cell applications.

### 3.5. Optical properties

**Fig. 9:**
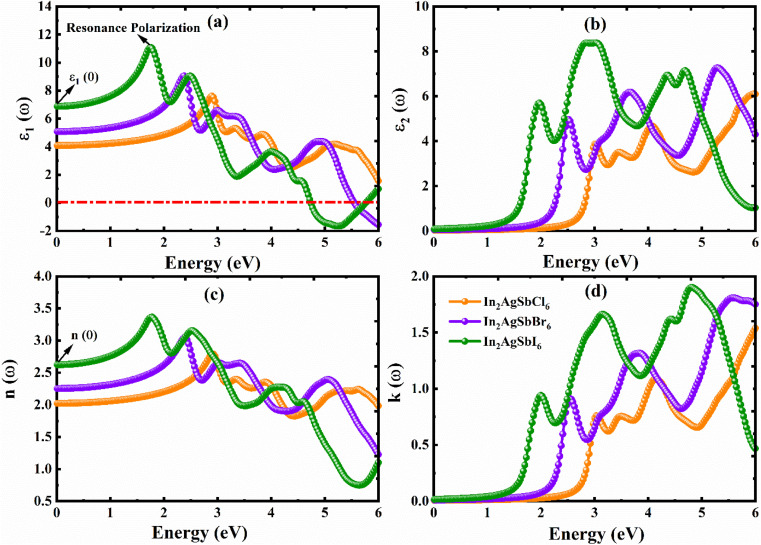
Computed parameters: (a) *ε*_1_(*ω*), (b) *ε*_2_(*ω*), (c) *n*(*ω*), and (d) *k*(*ω*).

**Fig. 10:**
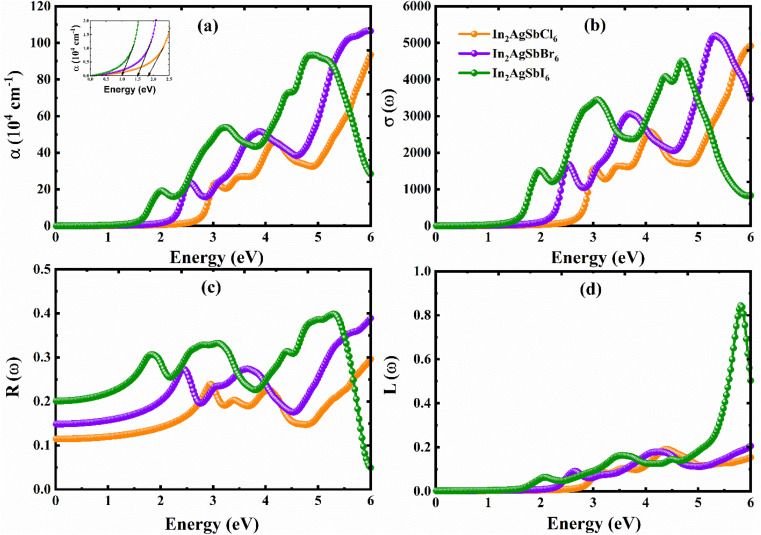
Computed parameters: (a) *α*(*ω*), (b) *σ*(*ω*), (c) *R*(ω), and (d) *L*(ω).

We can better comprehend materials' possible use in optoelectronics and photovoltaics by examining their optical characteristics, which offer important insights into how they respond to light. A thorough comprehension of a material's optical characteristics is necessary to maximize its capability in optoelectronics and solar cells. This work has thoroughly examined the optical characteristics of In_2_AgSbCl_6_, In_2_AgSbBr_6_, and In_2_AgSbI_6_. A medium's interaction with the incoming electromagnetic radiation is described by the dielectric function, *ε*(*ω*), which is defined *ε*(*ω*) = *ε*_1_(*ω*) + i*ε*_2_(*ω*). The *ε*(*ω*) can be used to determine optical characteristics by applying well-established mathematical concepts.^53–55^ All the optical parameters of In_2_AgSbCl_6_, In_2_AgSbBr_6_, and In_2_AgSbI_6_ were obtained between 0 and 6 eV, as shown in Fig. 9(a–d) and 10(a–d). The polarization behavior of a dielectric material is defined by the real part, *ε*_1_(*ω*). The absorptivity of halides can be described by the imaginary component, *ε*_2_(*ω*).^56^ To comprehensively understand the material's optoelectronic characteristics, *ε*_1_(*ω*) and *ε*_2_(*ω*) were computed as functions of photon energy in this DFT investigation. The *ε*_1_(*ω*), was calculated *via* the Kramers–Kronig transformation, and *ε*_2_(*ω*), was obtained by calculating the momentum matrix elements. All other optical characteristics were calculated using the *ε*_1_(*ω*) and *ε*_2_(*ω*) values of the dielectric function, as described in ref. 57 and 58.

Fig. 9(a) and (b) show the *ε*_1_(*ω*) and *ε*_2_(*ω*) values for In_2_AgSbX_6_ (X = Cl, Br, and I) under incident photon energies, respectively. The *ε*_1_(*ω*) curve starts at a static value, *ε*_1_(0), which is 4.08 for In_2_AgSbCl_6_, 4.67 for In_2_AgSbBr_6_, and 6.88 for In_2_AgSbI_6_, as shown in Fig. 9(a). The value of *ε*_1_(0) is important for determining the exciton binding energy (*E*^ex^_b_) required to separate electron–hole pairs. The materials with higher *ε*_1_(0) results in less *E*^ex^_b_, as In_2_AgSbI_6_ has higher *ε*_1_(0) among the studied substances and showed the lowest *E*^ex^_b_, as listed in Table 3. Moreover, the listed values for In_2_AgSbCl_6_, In_2_AgSbBr_6_, and In_2_AgSbI_6_ are considerably lower than those of CsPbI_3_ (0.068 eV),^50^ Cs_2_AgBiBr_6_ (0.268 eV),^51^ and Cs_2_AgBiBr_6_ (0.227 eV).^52^ These characteristics render In_2_AgSbCl_6_, In_2_AgSbBr_6_, and In_2_AgSbI_6_ for solar cells with an exceptional tendency. Then, with the increase in the photon energy, it climbs to its highest value, with an amplitude of 7.6 for In_2_AgSbCl_6_ at 2.90 eV, 9.1 for In_2_AgSbBr_6_ at 2.35 eV, and 11.1 for In_2_AgSbI_6_ at 1.75 eV. In the presence of photons with energies ranging from 4.7 to 6 eV, the halide DPs In_2_AgSbBr_6_ and In_2_AgSbI_6_ show negative values for *ε*_1_(*ω*), suggesting that both halides demonstrate total internal reflection of light in this region. According to this behavior, these materials appear to have significant reflectivity and low light transmission under photon energy settings.^59^ The fluctuating transformation rates of the incoming photons due to interaction with the DPs are responsible for the observed variations in *ε*_1_(*ω*).

Furthermore, inter-band transitions and intra-band transitions are two categories of transitions that determine a material's optical behavior. The metallic behavior of materials is characterized by intra-band transitions; thus, since the materials under study are semiconductors, only inter-band transitions were considered while determining the optical characteristics.^60^ The thresholds of *ε*_2_(*ω*) for In_2_AgSbCl_6_, In_2_AgSbBr_6_, and In_2_AgSbI_6_ correspond to electronic *E*_g_ (see Table 2), indicating that these DPs are semiconductors. All materials *ε*_2_(*ω*) start to rise as soon as their photon energy reaches the value of their individual electronic *E*_g_. In the electronic properties discussed above, the bandgap is correlated with the first absorption peak. The BS and DOS plots suggest the presence of deep energy states responsible for the secondary absorption peaks. These states result from orbital hybridization, as explained in ref. 61. For every material, the value of *ε*_2_(*ω*) increased with increasing photon energy until it reached its highest peak value (see Fig. 9(b)). According to the *ε*_1_(*ω*) and *ε*_2_(*ω*) results, the peak values for all materials were in the visible and UV areas.

The extinction coefficient, *k*(*ω*), and refractive index, *n*(*ω*), are two other significant optical characteristics that are crucial for assessing the energy gain and loss in materials during the manufacturing of optoelectronic memory devices.^57^ Through the following formulae,^61^*ε*(*ω*) is theoretically connected to *n*(*ω*), and *k*(*ω*):

6 *ε*_1_(*ω*) = *n*^2^ − *k*^2^

7 *ε*_2_(*ω*) = 2*nk*

Fig. 9(c and d) show the *n*(*ω*) and *k*(*ω*) values for In_2_AgSbX_6_ (X = Cl, Br, and I) under incident photon energies, respectively. The optical spectra of the materials under investigation are not well-documented either experimentally or theoretically. The *n*(*ω*) shows properties that are comparable to those of *ε*_1_(*ω*). The static *n*(0) values are 2, 2.3, and 2.6 for In_2_AgSbCl_6_, In_2_AgSbBr_6_, and In_2_AgSbI_6_. At photon energies of approximately 2.90 eV, 2.45, and 1.75 eV, the maximum recorded values of *n*(*ω*) for In_2_AgSbCl_6_, In_2_AgSbBr_6_, and In_2_AgSbI_6_ are 3.7, 3.1, and 3.4, respectively. The *n*(*ω*) graph shows a decreasing tendency with increasing photon energy after these peaks. The peak values for In_2_AgSbCl_6_, In_2_AgSbBr_6_, and In_2_AgSbI_6_ are 1.6, 1.7, and 1.9, respectively, at photon energies of 6 eV, 5.6 eV, and 4.9 eV. These *k*(*ω*) values are comparable to those of *ε*_2_(*ω*). According to *n*(*ω*) and *k*(*ω*) results, the peak values of all materials were in visible as well as UV areas.

The amount of light absorbed by a substance per unit length of propagation is measured by the absorption coefficient, *α*(*ω*).^62^ A restricted photon absorbing capacity is indicated by the transparency of materials with low *α*(*ω*). Materials with high *α*(*ω*), on the other hand, have efficient light absorbance capabilities. Consequently, *α*(*ω*) is a thorough indicator of how a material reacts to light. Fig. 10(a) demonstrates that no absorption occurs prior to the absorption edge; absorption only starts when the energy of the incident photons enters the visible spectrum. After the absorption edge, the *α*(*ω*) of these materials shows a notable increase, reaching peak magnitudes (×10^4^ cm) at energies of 3.08/4.1/6.0 eV for In_2_AgSbCl_6_, 2.5/3.9/5.9 eV for In_2_AgSbBr_6_, and 2.03/3.25/4.9 eV for In_2_AgSbI_6_. In the IR spectrum, they are transparent, whereas in the visible and UV regions, they efficiently absorb light. The intensities of the peaks in the vis-to-UV region were observed because more states were available for electronic transitions due to absorption.

The ability of a material to conduct electricity when exposed to light is referred to as photoconductivity or optical conductivity, *σ*(*ω*).^63^ This characteristic is essential for comprehending and creating optoelectronic devices. Fig. 10(b) shows the *σ*(*ω*) of In_2_AgSbX_6_ (X = Cl/Br/I) against photon energy. The peak values of all materials were observed at energies corresponding to *α*(*ω*). These variations in *σ*(*ω*) can be explained by stronger interactions between photons and the electronic structure of the material, as well as increased photon absorption. The increased photoconductive characteristics of In_2_AgSbX_6_ (X = Cl/Br/I) make it suitable for optoelectronic applications.

The capability of a material to bounce back light photons is measured by its optical reflectivity coefficient, *R*(*ω*).^64^Fig. 10(c) plots *R*(*ω*) against the photon energies of In_2_AgSbCl_6_, In_2_AgSbBr_6_, and In_2_AgSbI_6_. The static *R*(0) values were 0.12, 0.15, and 0.2 for In_2_AgSbCl_6_, In_2_AgSbBr_6_, and In_2_AgSbI_6_, respectively. The peak *R*(*ω*) values for In_2_AgSbCl_6_, In_2_AgSbBr_6_, and In_2_AgSbI_6_ were 0.24 at 3 eV, 0.36 at 5.5 eV, and 0.42 at 5.3 eV. These energy levels are within the ultraviolet (UV) range. These materials could therefore be very effective UV radiation shielding. Nevertheless, it is noted that for In_2_AgSbI_6_, *R*(*ω*) falls below 0.1 in the 5.5–6 eV energy range. The effective transparency of the incoming photons is indicated by low In_2_AgSbI_6_*R*(*ω*) within the range of the observed spectrum.

The loss function *L*(*ω*) measures the energy gained or lost during reflection and represents the energy loss in the medium.^63^ A material's energy loss function *L*(*ω*) is intimately related to *α*(*ω*) and *R*(*ω*). Since L(*ω*) is quite lower in magnitude for In_2_AgSbCl_6_, In_2_AgSbBr_6_, and In_2_AgSbI_6_ in the visible range of the energy spectrum; these halides exhibit high absorption. The *L*(*ω*) of In_2_AgSbCl_6_, In_2_AgSbBr_6_, and In_2_AgSbI_6_ are plotted in Fig. 10(d). In the energy range of 5.5–6 eV, the *L*(*ω*) value of In_2_AgSbI_6_ is approximately 0.89. In contrast, In_2_AgSbCl_6_ and In_2_AgSbBr_6_ exhibited a smaller increase in peak height. The higher *L*(*ω*) values suggest improved absorption capacities.

Consequently, the above discussion on parameters *ε*(*ω*), *α*(*ω*), and *σ*(*ω*) higher polarizability, absorption, and optical conduction in the visible regions for In_2_AgSbCl_6_, In_2_AgSbBr_6_, and In_2_AgSbI_6_. Moreover, *R*(*ω*) and *L*(*ω*) validates the effectiveness of these halides in visible regions with low reflective (<0.32) and optical losses (<0.2). Hence, In_2_AgSbCl_6_, In_2_AgSbBr_6_, and In_2_AgSbI_6_ are favorable materials as absorber layer materials in solar cells.

### 3.6. Thermoelectric (TE) properties

#### 3.6.1. TE parameters against temperature (*T*)

**Fig. 11:**
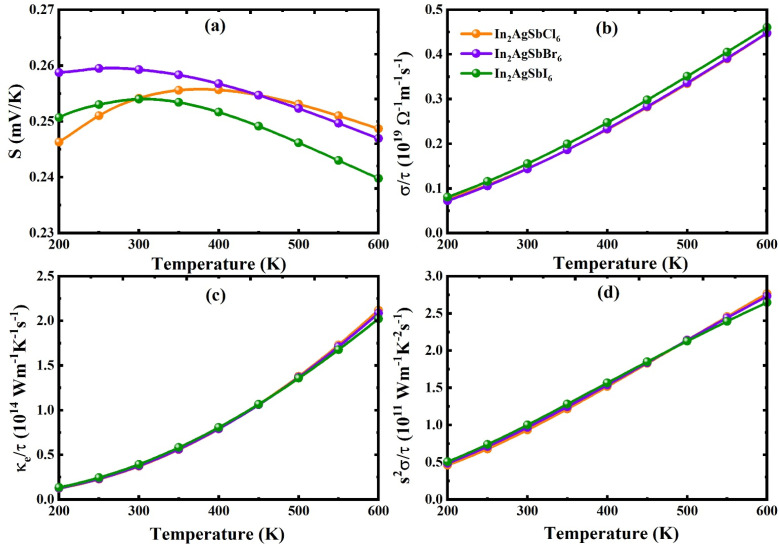
Plot of TE features varying with temperature: (a) *S* (b) *σ*/*τ* (c) *κ*_e_/*τ* (d) *S*^2^*σ*/*τ* for In_2_AgSbCl_6_, In_2_AgSbBr_6_, and In_2_AgSbI_6_.

The study of thermoelectric materials has recently attracted much attention. Using these substances, it is possible to convert wasted thermal energy into usable energy. Solar panels, electrical generators, and heat transmission are some of the many uses of thermoelectric substances.^65^ The *ZT* measurement for thermoelectric substances must be equal to at least one for them to be used in real-world applications. The power factor, which incorporates both the Seebeck coefficient (*S*) and electrical conductivity (*σ*), and the capability of the material to efficiently transmit heat, determines a material's competence as a source of thermoelectric power. A material's capacity to capture energy is determined by its appropriate bandgap, which is an essential property for energy storage applications. Because of its capacity to transform heat into energy, TE materials have attracted much interest worldwide. The TE properties of In_2_AgSbCl_6_, In_2_AgSbBr_6_, and In_2_AgSbI_6_ were elucidated in detail as a function of temperature fluctuations. For In_2_AgSbCl_6_, In_2_AgSbBr_6_, and In_2_AgSbI_6_, Fig. 11(a–d) presents the computed thermal transport attributes over the 200–600 K temperature range.

The potential difference caused by a temperature gradient across the material is measured by *S*, which is an important parameter.^66^ The predominant charge carrier is determined by the Seebeck coefficient (*S*), which also helps determine the voltage generated by temperature fluctuations. Charge carriers are classified according to their sign, where positive and negative S denote holes and electrons, respectively. The p-type semiconductor nature of these materials is indicated by the positive values of *S* (see Fig. 11(a)), which confirms that the majority of charge carriers in In_2_AgSbX_6_ (X = Cl/Br/I) are holes. When *T* was increased to 600 K, the *S* values of all materials decreased. *S* decreased from 0.254, 0.259, and 0.254 (in mV K^−1^) at 300 K to 0.248, 0.247, and 0.240 (in mV K^−1^) for In_2_AgSbCl_6_, In_2_AgSbBr_6_, and In_2_AgSbI_6_, respectively.

The electrical conductivity (*σ*/*τ*) is another crucial TE characteristic that quantifies how the electric current flows as temperature changes.^67^ Due to increased mobility and carrier concentration at higher temperatures, electrons can move through the material more readily, as seen by the *σ*/*τ* (see Fig. 11(b)), increasing linearly with temperature. Due to Ag's lower electronegativity than Sb, a notable improvement in *σ*/*τ* is shown. Higher carrier concentration, enhanced charge mobility, and decreased power consumption are the outcomes of Ge's lower electronegativity, which causes valence electrons to be bound loosely. The *σ*/*τ* demonstrates the maximum intensities (×10^19^ Ω^−1^ m^−1^ s^−1^) of 0.44, 0.45, and 0.46 for In_2_AgSbCl_6_, In_2_AgSbBr_6_, and In_2_AgSbI_6_, respectively.

The material's heat capacity is indicated by its thermal conductivity (*κ*_e_/*τ*).^67^ A greater density of states (DOS) close to the Fermi level is linked to higher *κ*_e_/*τ*, which enhances *σ*/*τ* by offering additional energy levels for electron mobility. To sustain a high-temperature gradient and achieve the best TE capability, low *κ*_e_/*τ* is necessary (see Fig. 11(c)). According to the theoretical predictions, all materials exhibit an increase in *κ*_e_/*τ* as the temperature rises, indicating efficient heat transport. Similar to *σ*/*τ*, *κ*_e_/*τ* rises with temperature, and for In_2_AgSbCl_6_, In_2_AgSbBr_6_, and In_2_AgSbI_6_ have *κ*_e_/*τ* of 1.24, 1.27, and 1.35 × 10^14^ W m^−1^ K^−1^ s^−1^ at 200 K, whereas they are 2.11, 2.08, and 2.02 × 10^14^ W m^−1^ K^−1^ s^−1^ at 600 K, respectively.

The power factor (PF) of a material, which is crucial for assessing TE capability, is computed as PF = *S*^2^*σ*/*τ*, where *σ*/*τ* is the electrical conductivity and S is the Seebeck coefficient.^64^ At 200 K, the PF starts at 0.46, 0.48, and 0.51 × 10^11^ W m^−1^ K^−2^ s^−1^ for In_2_AgSbCl_6_, In_2_AgSbBr_6_, and In_2_AgSbI_6_, respectively. At 600 K, the values reached 2.76, 2.71, and 2.65 × 10^11^ W m^−1^ K^−2^ s^−1^, respectively (Fig. 11(d)). These results indicate that all materials are suitable for high-temperature applications because their PFs steadily increase with temperature.

#### 3.6.2. TE parameters against chemical potential (CP)

**Fig. 12:**
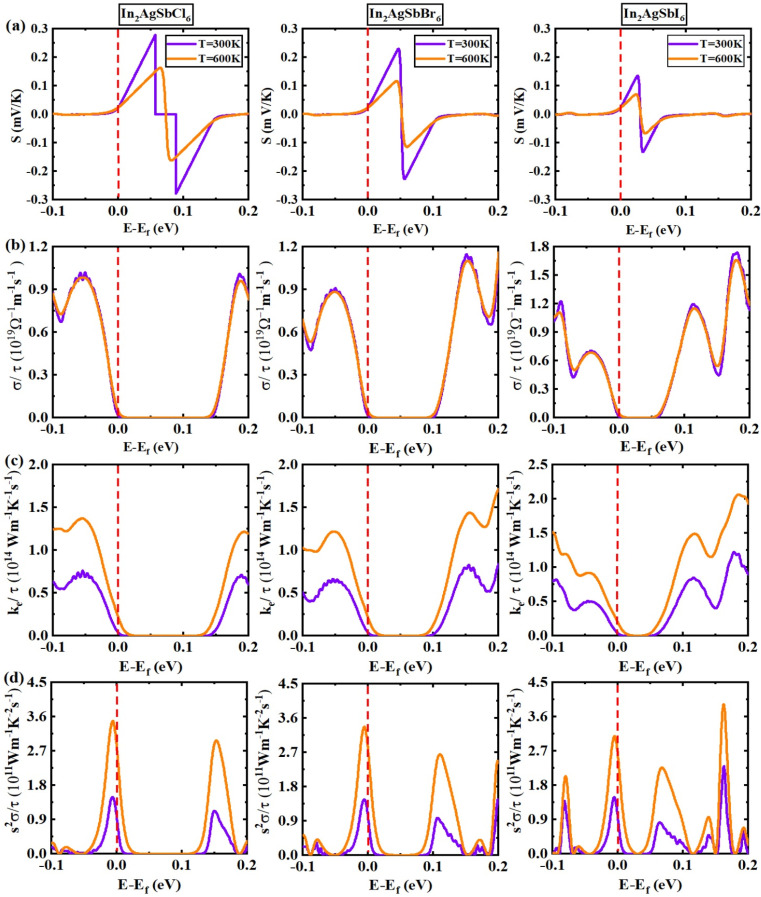
TE parameters against CP = *E* − *E*_f_ (eV): (a) *S* (b) *σ*/*τ* (c) *κ*_e_/*τ* (d) *S*^2^*σ*/*τ*.

The thermal transport characteristics of In_2_AgSbCl_6_, In_2_AgSbBr_6_, and In_2_AgSbI_6_ are shown to vary with the chemical potential (CP = *E* − *E*_f_ (eV)) in Fig. 12(a–d) for temperatures of 300 K and 600 K. Fig. 12(a) shows that *S* is dependent on the CP in the range of −1.0–2.0 eV. Within the investigated CP range, p-type and n-type behaviors can be observed. The best *S* values were between 0.0 eV and 0.1 eV for In_2_AgSbCl_6_ and In_2_AgSbBr_6,_ whereas they were between 0.0 eV and 0.05 eV for In_2_AgSbI_6_. In addition, In_2_AgSbCl_6_, In_2_AgSbBr_6_, and In_2_AgSbI_6_ did not show any significant peaks between −0.1 and 0.0 eV (Fig. 12(a)). The peak values in the p-type region confirm the p-type semiconducting features^68^ of In_2_AgSbCl_6_, In_2_AgSbBr_6_, and In_2_AgSbI_6_. In addition, for In_2_AgSbCl_6_, In_2_AgSbBr_6_, and In_2_AgSbI_6_, the maximum S value decreased as the temperature increased from 300 K to 600 K. An electrical current is generated as electrons move from a hot region to a cold region. High conductivity is an important property of high-quality TE materials. At 300 K and 600 K, the electrical conductivities for In_2_AgSbCl_6_, In_2_AgSbBr_6_, and In_2_AgSbI_6_ are shown in Fig. 12(b), revealing their TE capability. At 300 K, the highest magnitudes for n-type In_2_AgSbCl_6_, In_2_AgSbBr_6_, and In_2_AgSbI_6_ are 0.98 (−0.052 eV), 0.88 (−0.049 eV), and 1.10 (−0.093 eV) × 10^19^ Ω m^−1^ s^−1^, respectively, which slightly rises at 600 K. Meanwhile, the highest peak values for p-type are observed as 0.95 (0.19 eV), 1.10 (0.15 eV), and 1.67 (0.18 eV) × 10^19^ Ω m^−1^ s^−1^, respectively, at 300 K, which show negligible increase at 600 K. The increase in electrical conductivity can be attributed to a direct correlation with the charge carrier density.^69^Fig. 12(c) also shows the change in *κ*_e_/*τ* with CP from −1.0 eV to 2.0 eV at 300 K and 600 K, which shows a growing trend with increasing temperature. Fig. 12(d) demonstrates that the power factor (PF), which has been investigated for CP ranging from −1.0 eV to 2.0 eV at temperatures of 300 K and 600 K, reaches a peak value near the Fermi level for In_2_AgSbCl_6_, In_2_AgSbBr_6_, and In_2_AgSbI_6_, suggesting the p-type nature of these semiconductors. In addition, as demonstrated in Fig. 12(d), the intensity of the PF against CP increased with increasing temperature. Although it rose with increasing temperature, the maximum value was close to the Fermi level. Therefore, this analysis clarifies that In_2_AgSbCl_6_, In_2_AgSbBr_6_, and In_2_AgSbI_6_ are semiconductors with a p-type nature and have appealing features for use in TE technologies.^70^

#### 3.6.3. Figure of merit against temperature

When assessing the TE effectiveness of a material, *ZT*—also called the TE figure of merit—is a crucial metric to consider. TE materials with higher *ZT* values are preferable because they transform thermal energy more efficiently into electric power with less heat loss. If *ZT* is one or greater, the device is not an efficient TE device. The value of *ZT* can be found using its dimensionless form in the following formula:^71^8
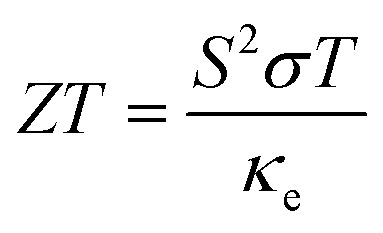


**Fig. 13:**
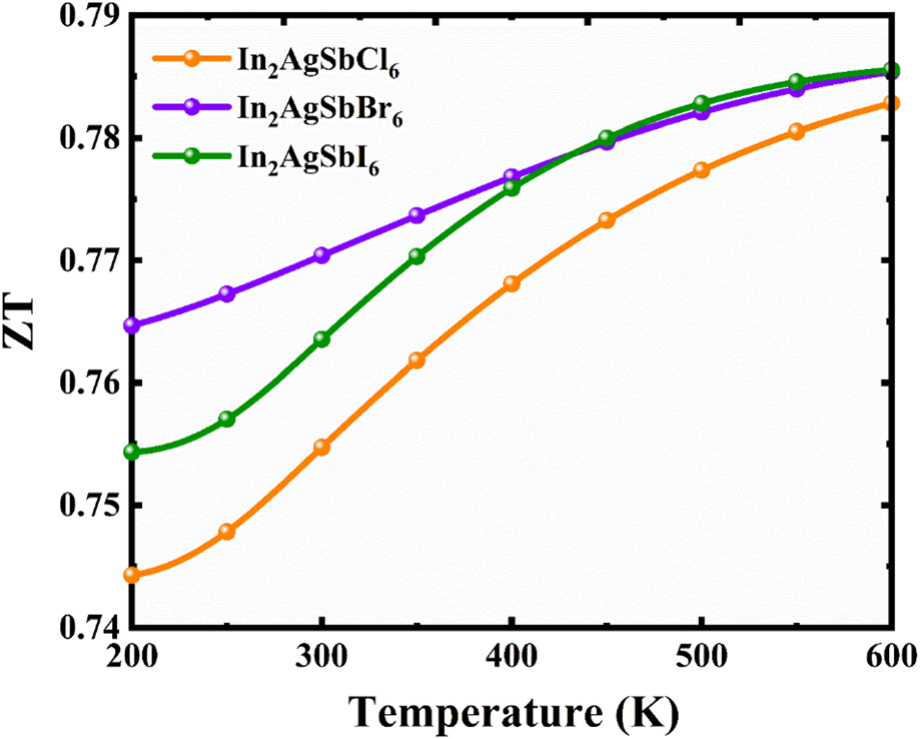
TE figure of merit in relation to the T for In_2_AgSbCl_6_, In_2_AgSbBr_6_, and In_2_AgSbI_6_.

For the practical application of substances in TE technologies, parameters *S* and *σ* should be increased because these features directly affect the TE performance. The thermal conductivity (*κ*_e_/*τ*) impacts inversely on the TE performance and should be lower than the *S* and *σ*. At 300 K (room temperature), the *ZT* values of In_2_AgSbCl_6_, In_2_AgSbBr_6_, and In_2_AgSbI_6_ were 0.75, 0.77, and 0.76, respectively. Fig. 13 shows similar plots for In_2_AgSbCl_6_, In_2_AgSbBr_6_, and In_2_AgSbI_6_, with *ZT* increasing from 200 K to 600 K. The highest values for these DP compounds were 0.785 for In_2_AgSbCl_6_, In_2_AgSbBr_6_, and 0.780 for In_2_AgSbI_6_. These values demonstrate efficient thermal energy conversion into useful power at room temperature and higher temperatures.

Additionally, these substances offer higher *ZT* values than several reported similar halides, such as Cs_2_AgBiCl_6_ (0.72), Cs_2_AgBiBr_6_ (0.71),^72^ Cs_2_AgSbCl_6_ (0.72), and Cs_2_AgSbBr_6_ (0.73),^73^ at 300 K. Consequently, the elevated *ZT* measurements for In_2_AgSbCl_6_, In_2_AgSbBr_6_, and In_2_AgSbI_6_ indicate that it is capable of effectively converting thermal radiation into electricity, rendering it an appealing choice for thermal energy recovery devices and the production of thermal electricity. The prospective use of the substance in TE devices is further supported by the substantial Seebeck coefficient, which measures the voltage produced in reaction to a difference in temperature across the material, demonstrating a robust TE effect. In_2_AgSbCl_6_, In_2_AgSbBr_6_, and In_2_AgSbI_6_ are advantageous materials for the development of thermoelectric systems for a variety of manufacturing purposes due to their elevated *ZT* and *S* values. These results suggest that TEs can achieve superior performance. Therefore, it provides noteworthy energy transmission features.

## 4. Conclusion

Briefly, the stability, optoelectronic, and thermal transport characteristics of In_2_AgSb(Cl/Br/I)_6_ were investigated by comprehensive DFT exploration. A tolerance factor ranging between 0.94 and 0.95 suggests cubic stability and a formation energy ranging between −1.97 and −1.32 eV per atom ensured thermal stability. The AIMD analysis confirmed the dynamic stability of In_2_AgSb(Cl/Br/I)_6_ up to 700 K. The mechanical stability and asymmetric and ductile characteristics validated suitability in foldable technologies. The electrical properties revealed indirect band gaps (*E*_g_) of 1.95 eV, 1.35 eV, and 0.78 eV for In_2_AgSbCl_6_, In_2_AgSbBr_6_, and In_2_AgSbI_6,_ respectively, which lie in the acceptable region for solar cell applications. The lower binding energies of excitons for In_2_AgSbCl_6_, In_2_AgSbBr_6_, and In_2_AgSbI_6_ compared with the efficient solar cell materials CsPbI_3_ and Cs_2_AgBiBr_6_ confirmed their effectiveness as absorber layer materials. The optical analysis revealed a higher dielectric constant and absorption, whereas low reflection and loss of photons during interaction in the visible and ultraviolet spectrum. The thermal transport response exhibited a p-type nature, with higher *S* and *ZT* values of 0.75, 0.77, and 0.76, respectively. These characteristics demonstrate that In_2_AgSbCl_6_, In_2_AgSbBr_6_, and In_2_AgSbI_6_ are effective options for future energy harvesting technologies.

## Data availability

The data will be made available on request.

## Conflicts of interest

The authors declare no competing interests.
